# Seeding of proteins into amyloid structures by metabolite assemblies may clarify certain unexplained epidemiological associations

**DOI:** 10.1098/rsob.170229

**Published:** 2018-01-24

**Authors:** Dorin Sade, Shira Shaham-Niv, Zohar A. Arnon, Omid Tavassoly, Ehud Gazit

**Affiliations:** 1 Department of Molecular Microbiology and Biotechnology, Tel Aviv University, Tel Aviv 6997801, Israel; 2 Sagol Interdisciplinary School of Neurosciences, Tel Aviv University, Tel Aviv 6997801, Israel; 3 Blavatnik Center for Drug Discovery, Tel Aviv University, Tel Aviv 6997801, Israel; 4 Department of Chemistry, Simon Fraser University, Burnaby, British Columbia, Canada V5A 1S6

**Keywords:** amyloid seeding, inborn error of metabolism, mechanism of neurodegeneration, metabolite accumulation, metabolite amyloids, oncometabolites

## Abstract

The accumulation of various metabolites appears to be associated with diverse human diseases. However, the aetiological link between metabolic alteration and the observed diseases is still elusive. This includes the correlation between the abnormally high levels of homocysteine and quinolinic acid in Alzheimer's disease, as well as the accumulation of oncometabolites in malignant processes. Here, we suggest and discuss a possible mechanistic insight into metabolite accumulation in conditions such as neurodegenerative diseases and cancer. Our hypothesis is based on the demonstrated ability of metabolites to form amyloid-like structures in inborn error of metabolism disorders and the potential of such metabolite amyloids to promote protein aggregation. This notion can provide a new paradigm for neurodegeneration and cancer, as both conditions were linked to loss of function due to protein aggregation. Similar to the well-established observation of amyloid formation in many degenerative disorders, the formation of amyloids by tumour-suppressor proteins, including p53, was demonstrated in malignant states. Moreover, this new paradigm could fill the gap in understanding the high occurrence of specific types of cancer among genetic error of metabolism patients. This hypothesis offers a fresh view on the aetiology of some of the most abundant human maladies and may redirect the efforts towards new therapeutic developments.

## 1. Metabolite accumulation and amyloid-like structure formation

**Figure 1.**
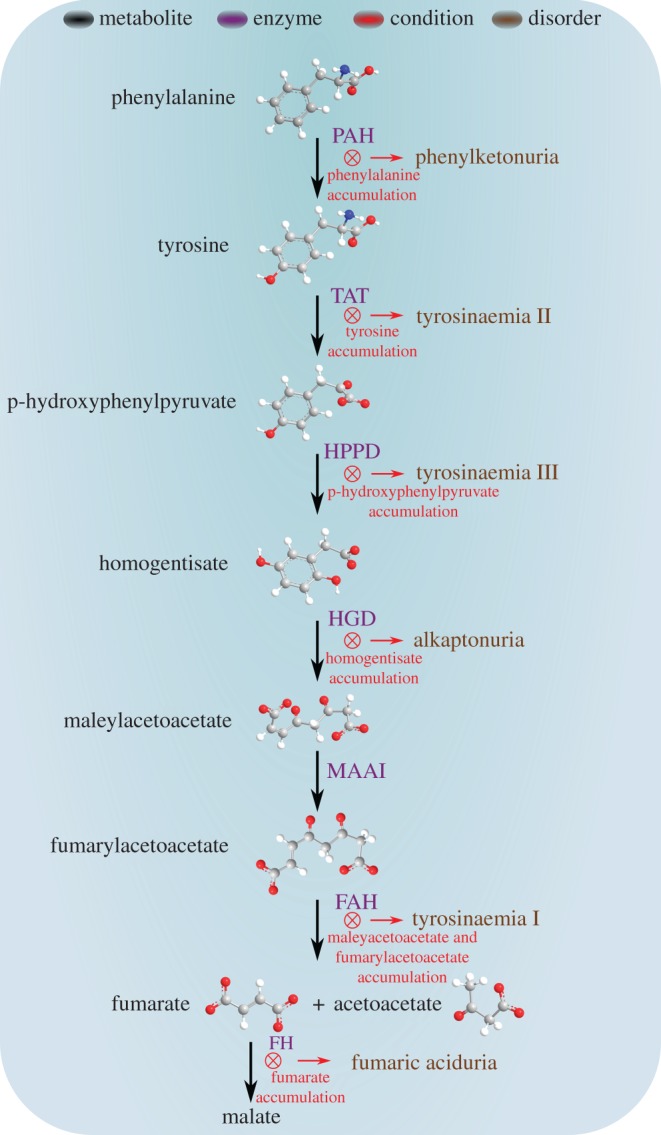
Scheme of the biosynthetic pathway downstream to phenylalanine. When enzyme deficiencies occur, specific metabolites accumulate (denoted in red), leading to particular disorders (denoted in brown). PAH, phenylalanine hydroxylase; TAT, tyrosine aminotransferase; HPPD, ρ-hydroxyphenylpyruvate dioxygenase; HGD, homogentisate 1,2-dioxygenase; MAAI, maleylacetoacetate isomerase; FAH, fumarylacetoacetase; FH, fumarate hydratase.

Many diseases stem directly from variation in activity, folding and stability of proteins. While the role of altered protein function in diseases is well established, an important aspect associated with failures in biosynthetic pathways is often overlooked. Interference in a given metabolic process can lead to deficiencies or accumulation of metabolites (figure 1). This can, in turn, affect multiple biological functions, including signalling, structural organization, stimulation and inhibitory processes. Metabolites, such as amino acids, nucleobases, neurotransmitters, organic acids and their downstream intermediates and products, play an important part in cellular homeostasis. However, in many cases, the roles of metabolites are not fully understood [1,2].

The maintenance of metabolite homeostasis is an important part of cellular physiology, yet the accumulation of metabolites was observed in various diseases when phenotypic variations occur. Inborn error of metabolism (IEM) disorders, a group of well-known genetic diseases, are induced by mutations resulting in the malfunction of specific enzymes, leading to disrupted biosynthetic pathways and accumulation of metabolites. The accumulated metabolites can be toxic and interfere with the normal function of cells and tissues [3,4]. For example, in phenylketonuria (PKU) patients, phenylalanine accumulates in the plasma, cerebrospinal fluid and brain tissue due to a mutation in the gene encoding for phenylalanine hydroxylase (PAH) (figure 1) [5,6]. Moreover, several other IEM disorders result from downstream blockades in this biosynthetic pathway, as illustrated in figure 1. Individuals with such disorders can show severe symptoms, including mental retardation, epilepsy, organ damage and other developmental abnormalities, which can be avoided only with a strict diet [1,7]. It should be noted that such mutations can lead to very high concentrations of the metabolites. For example, in PKU, the blood concentration of phenylalanine can reach values over 1.2 mM [8,9] in untreated patients as compared with 35–85 µM in healthy individuals.

Another characteristic of metabolites, which until recently was attributed solely to proteins and peptides, is their ability to form ordered amyloid-like assemblies [10–13]. Amyloid-associated diseases, in which proteins and polypeptides form ordered aggregated assemblies, are a group of very common degenerative disorders, including Alzheimer's disease (AD), Parkinson's disease (PD), amyotrophic lateral sclerosis (ALS) and type II diabetes. The fibrillary deposits in these pathological conditions are located in the intracellular or extracellular milieu of various organs and tissues, where they may induce apoptotic cell death [14,15]. In recent studies, it was established that under physiological conditions, various metabolites associated with IEM disorders could form ordered structures that highly resemble protein amyloid assemblies [16–22]. Specifically, phenylalanine, adenine, orotic acid, cystine, tyrosine, tryptophan, glycine, histidine and uracil, which individually accumulate in specific IEM disorders (table 1), were found to possess the capability to form amyloid-like structures. The metabolite amyloids have a clear fibrillary structure, bind to amyloid-specific dyes and show a dose-dependent apoptotic effect [11–13]. Using centrifugation to pellet the structures from solutions, it was confirmed that the toxic effect is due to the formation of supramolecular metabolite structures, rather than the concentration of metabolite monomers [12]. Furthermore, phenylalanine fibrils could be specifically detected using antibodies raised against the formed structures, another amyloid-like property [13,37]. Thus, it can be hypothesized that part of the pathologies reported in IEM disorders are a result of metabolite accumulation and amyloid formation. This hypothesis was further supported by demonstrating the presence of phenylalanine deposits in post-mortem brain sections of PKU patients using immunohistochemistry and Congo red staining [13].

**Table 1.** Metabolites that accumulate in human diseases.

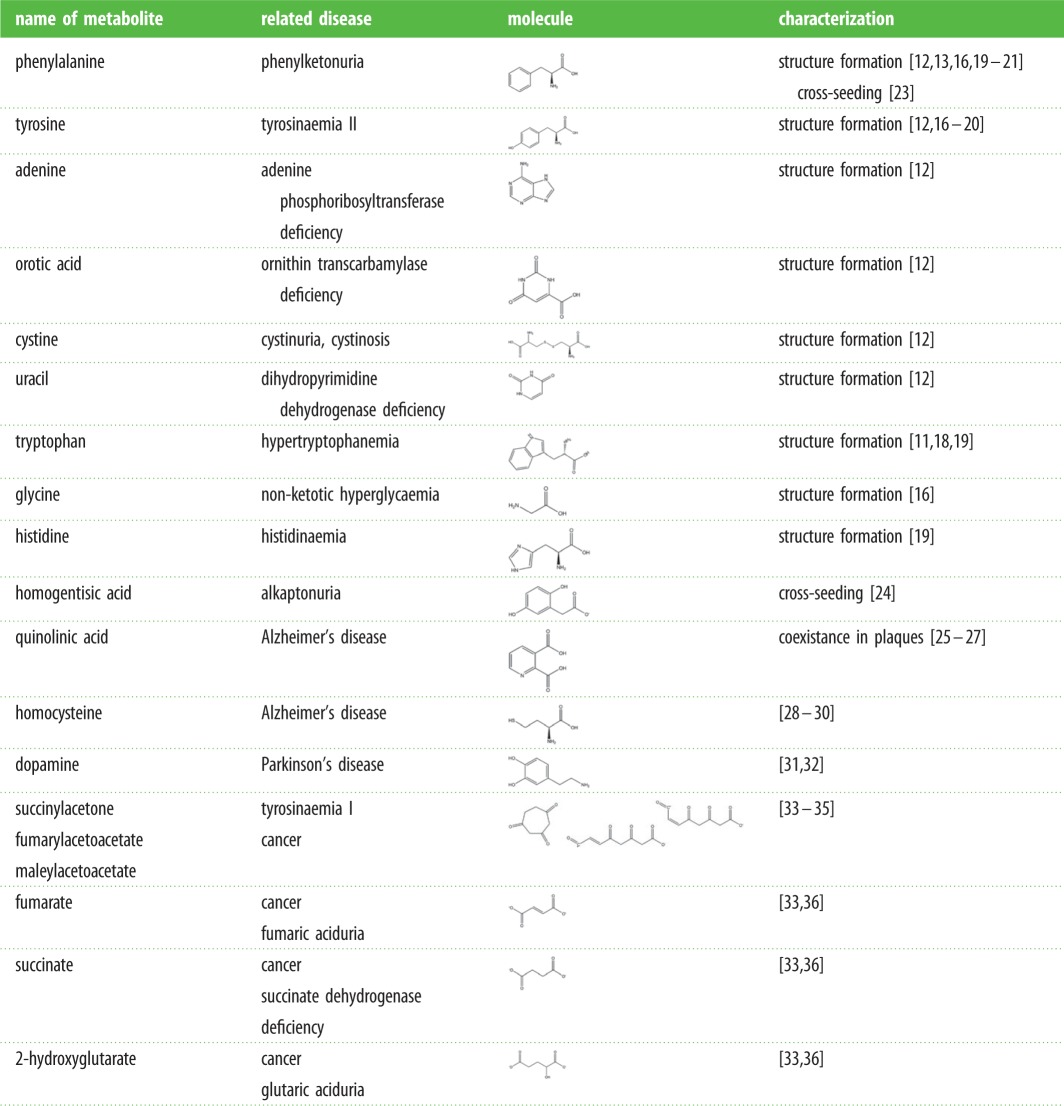

## 2. Seeding of proteins by metabolite assemblies

Recent studies demonstrated that metabolites could cross-seed proteins [23]. Phenylalanine formed fibrils initiated the aggregation of several non-amyloidogenic proteins under physiological conditions [23]. Globular proteins, including lysozyme, serum albumin, insulin, myoglobin and cytochrome *c*, spontaneously self-assembled into amyloid fibrils in the presence of phenylalanine seeds [23]. Another observation was the accelerated aggregation of a soluble mixture of amino acids following the addition of phenylalanine fibrils [23]. Additional study demonstrated the cross-seeding of proteins by homogentisic acid (HGA), a metabolite related to alkaptonuria [24], a rare IEM disorder which was lately classified as secondary amyloidosis. Alkaptonuria is characterized by the accumulation of HGA due to deficiency of homogentisate 1.2-dioxygenase enzyme (figure 1, table 1). HGA was shown to induce the aggregation and fibrillization of amyloidogenic proteins, such as serum amyloid A, β-amyloid polypeptide (Aβ) and α-synuclein. HGA was suggested to be an important amyloid co-component in alkaptonuria amyloidosis [24]. Self- and cross-seeding of proteins by amyloid assemblies is well established in the literature [28,38], and we propose to extend this concept to include metabolite assemblies as possible seeds that may be formed upstream to protein aggregation and may facilitate this process.

**Figure 2.**
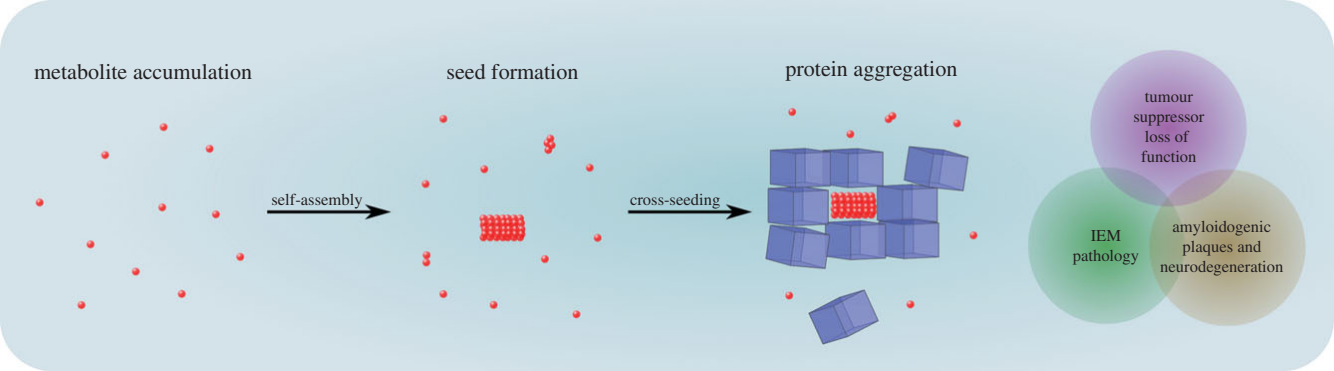
Metabolite seeding hypothesis. A schematic putative model for the seeding of proteins by metabolite assemblies. Accumulated metabolites self-assemble into ordered structures. In turn, the structures serve as seeds to increase further aggregation of proteins. Loss of function of different proteins induces various pathological effects, as shown on the right.

This ability of simple metabolites to form ordered structures, and the seeding effect demonstrated by phenylalanine and HGA, provides new paradigm for numerous diseases that could be tested experimentally. Structure formation and cross-seeding of proteins may be a possible mechanism in which accumulated metabolites interfere with protein function and folding (figure 2). Disrupting the function and folding of tumour-suppressor proteins, such as p53, or amyloidogenic proteins, such as α-synuclein, Aβ and tau, may induce corresponding pathological effects. We propose that metabolite accumulation and molecular self-assembly can be the early event in a cascade that leads to neurodegeneration and malignant processes.

Indeed, the accumulation of metabolites was extensively associated not only with IEM disorders but also with other human diseases. Many observations, which will be discussed below, support the concept that metabolites might play significant roles in these major epidemiological maladies. Briefly, the accumulation of several metabolites was demonstrated to be involved in AD [25–27,29,30,39–42] (table 1). Quinolinic acid (QA), an endogenous metabolite, was shown to accumulate in amyloid plaques [29,30,40] and to affect tau protein aggregation [39]. Homocysteine (Hcy), a non-coded amino acid, was identified as a risk factor in AD due to its high concentration in the plasma of patients and its cytotoxicity effect on hippocampal and cortical neurons [25,41,42]. In addition, few studies demonstrated that dopamine, which is strongly related to PD, induces α-synuclein aggregation into soluble oligomers [33,36,43]. Likewise, accumulation of several metabolites was linked with increased cancer risk. Fumarate, succinate and 2-­hydroxyglutarate (2HG) (table 1) have been described as ‘oncometabolites’ that promotes malignancy [44,45]. Furthermore, hepatocellular carcinoma (HCC), a common cancer type among IEM disorders patients [46], may be induced by metabolite accumulation [45].

Taken together, in addition to IEM disorders, metabolites might also play a significant role in cancer and neurodegeneration. However, the specific role of metabolites in the pathology of these diseases is still elusive. We suggest metabolite assembly and cross-seeding following accumulation as a fundamental mechanism that might explain some unidentified epidemiological aspects associated with pathological conditions (figure 2).

## 3. Metabolite accumulation in neurodegeneration

QA is an endogenous metabolite that is involved in the pathology of neurodegenerative diseases [40,47,48] (table 1). It is a downstream product of the kynurenine pathway, the primary route of tryptophan degradation in mammalian cells. QA is produced by macrophages and activated microglia. Under normal conditions, the metabolite is catabolized by the quinolinate phosphoribosyltransferase (QPRTase) enzyme to maintain its cellular levels at very low concentrations [49–51]. While neurons are unable to synthesize QA, intracellular QPRTase has been detected in these cells [52,53]. Under pathological conditions, due to inflammatory responses, the kynurenine pathway is over-activated and the production of QA increases [54,55]. Excess QA can be internalized by neurons in an unknown molecular mechanism [29,39], resulting in saturated QPRTase activity and QA accumulation outside and inside neurons [39,56]. In post-mortem brain sections of AD patients, intracellular QA has been detected as punctate structures, in co-localization with tau protein fibrillary structures [39,48]. It has been demonstrated that treatment of primary cultures of human neurons with QA increases both total and phosphorylated tau [39]. Interestingly, QPRTase knock-out has been identified to significantly increase Aβ accumulation in mouse brain [57], highlighting the importance of QPRTase in accumulation of aggregated proteins. This suggests that QA accumulation may be an important factor in the complex cascade that eventually leads to neurodegeneration [48,56]. We hypothesize that excess QA accumulation may lead to metabolite amyloid-like fibril formation, which cannot be degraded by QPRTase. The putative QA fibrils co-localize with aggregated endogenous proteins, and may serve as a seed to increase further aggregation of pathological, aggregation-prone proteins, such as tau, Aβ and α-synuclein (figure 2).

Accumulation of Hcy was identified as a clear risk factor in AD (table 1). High levels of Hcy in the plasma (denoted as hyperhomocysteinaemia or HHcy) were observed in AD patients [25] and are associated with markers of AD. For example, as determined by MRI, the hippocampal and cortical volume of patients decreased significantly with increasing Hcy plasma concentration [58]. Furthermore, Hcy-rich medium was shown to be cytotoxic to hippocampal and cortical neurons, resulting in increased Aβ-induced cell death [41,42]. Furthermore, HHcy induced in the brains of AD transgenic mouse models caused an elevation of A*β* deposition. Hcy was found to bind A*β*_40_, thereby stimulating a β-sheet structure formation to facilitate its deposition [31,32,59]. Recently, it was reported that Hcy can induce hyperactivity of a key kinase, the mechanistic target of rapamycin complex 1 (mTORC1), a newly identified risk factor for sporadic AD. The mTORC1 hyperactivity can inhibit neuronal clearance and autophagy pathways, leading to abnormal Aβ and phosphorylated tau accumulation and aggregation [27]. We speculate that Hcy might undergo fibril formation and induce the seeding of Aβ polypeptide resulting in plaque formation (figure 2).

Furthermore, a recent report suggested another possible link between metabolite assembly and amyloid formation in PD. It was shown that increased dopamine levels induce the formation of toxic oligomers of mutated α-synuclein [33]. While alternative models that are associated with neuromelanin formation were suggested, the seeding of α-synuclein by dopamine supramolecular species cannot be ruled out. This may explain the observation that neuromelanin polymers do exist in healthy individuals with no PD symptoms. Taken together, the metabolite seeding hypothesis (figure 2) should be tested experimentally to assess the possible link, based on indications found in the literature for dopamine accumulation [34,35].

## 4. Oncometabolite accumulation in cancer

Metabolic dysregulation by IEM disorders is associated with cancer, as lately reviewed by Erez *et al.* [45]. Several disorders associated with a single gene mutation leading to enzymes deficiency have been linked with increased cancer risk, and the accumulation of toxic metabolites has been described as a major event in the pathway to malignancy [45]. For example, Tyrosinaemia type I is the result of deficiency in fumarylacetoacetase (FAH), the last enzyme of the tyrosine degradation pathway (figure 1, table 1). As a result, the accumulation of the toxic metabolites succinylacetone, fumarylacetoacetate and maleylacetoacetate toxic metabolites can cause tissue damage, fibrosis and cirrhosis when taking place in the liver [60,61]. These conditions can promote malignant processes, and were specifically connected to HCC, a common cancer type among IEM disorder carriers [46]. Preventing the accumulation of toxic metabolites using nitisinone, an inhibitor of ρ-hydroxyphenylpyruvate dioxygenase (figure 1), showed to reduce cancer risk in tyrosinaemia type I patients and improve organ function. Induced HCC by metabolite accumulation was also observed in haemochromatosis, porphyria and Wilson disease, and other cancer types, such as renal cell carcinoma and haematological cancers, were shown to be associated with Fabry disease and Gaucher disease, respectively [45,61]. All of these diseases are IEM disorders that include chronic and toxic accumulation of metabolites. Furthermore, several other metabolites have been described as ‘oncometabolites’ that promote malignancy upon accumulation. For example, fumarate, succinate and 2HG (table 1), accumulate due to mutations in the genes encoding fumarate hydratase (FH), succinate dehydrogenase and isocitrate dehydrogenase 1 or 2 (IDH1/2), respectively, enzymes of the tricarboxylic acid cycle [45]. These oncometabolites were demonstrated to alter the activity of proteins and transcription factors, leading to dramatic remodelling of gene expression pattern and DNA epigenetic modification. Such alteration can promote DNA damage, oncogenic cell survival and proliferation, formation of blood vessels and impairment of cellular differentiation, all characteristics of tumour development and malignancy [44,45].

We hypothesize that these oncometabolites, like other metabolites mentioned, could also undergo self-assembly into ordered structures, which can interfere with the function of cells. One possible interference is the seeding of tumour-suppressor proteins such as p53 and VHL [62,63], both demonstrated to have marginal stability and to be prone to aggregation and amyloid formation, which in turn results in their loss of function [64–66] (figure 2). However, the molecular basis of the aggregation of tumour-suppressor proteins is still not fully understood. Our hypothesis could provide a new direction for elucidating this observation.

## 5. Concluding remarks

Metabolite self-assembly and cross-seeding of proteins should be further investigated as it may serve as new target for therapy. The accumulation of metabolites and alterations in specific metabolic pathways may contribute to many pathological effects [2–4] (figure 2) and thus should be specifically monitored using high-throughput ‘metabolomics’ approaches. Recent studies of metabolite profiling in body fluids and brain regions have revealed alterations in specific metabolic pathways in a set of different diseases. For example, phenylalanine, tryptophan and tyrosine metabolism is commonly altered in PD, ALS, PKU and Huntington's disease, and metabolism of the first two is also altered in AD [67–70]. This observation suggests that shared pathological symptoms may be the result of accumulation and structure formation of these aromatic amino acids. There are also indications for alterations in glycine and histidine metabolism in PD and ALS, respectively [70]. Indeed, these amino acids were shown to accumulate in IEM disorders and to form structures (table 1) [12,16,19]. Specifically for AD and PD, it was observed in many metabolomics studies that the kynurenine pathway of tryptophan metabolism is increased [67,71], consistent with the observations of QA (a downstream intermediate) accumulation in amyloid plaques [30]. This provides further support to our hypothesis, that QA accumulation may be toxic and alters protein function, leading to neuronal dysfunction (figure 2). Another important direction is the cross analysis of information from genetic studies. Such studies demonstrated that aminocarboxymuconate semialdehyde decarboxylase (ACMSD), a key enzyme in the kynurenine pathway is associated with PD [72]. Furthermore, mutations in ACMSD cause an increase in the level of QA [73–76]. Taken together, ACMSD and QPRTase enzymes deficiencies may lead to very high concentrations of QA, similar to IEM disorders. Therefore, the accumulation of QA as well as other metabolites that are mentioned here should be further examined using metabolomics approaches.

Finally, while *a priori* it appears counterintuitive that metabolites could form ordered and stable structures that could seed much larger biomolecules, it should be remembered that aggregation into ordered macroscopic structures is well known in the formation of gallstone, kidney stones and gout-related crystals [77]. Therefore, very simple metabolites contain all the molecular information needed to form stable and well-ordered structures. Since no clear similarity between the various amyloid-forming metabolites is evident, additional metabolites could have the potential to form such structures. This resembles the ability of numerous non-disease-related proteins to form amyloid structures and the recognition of the amyloid as a generic organization of proteins [78–81]. Thus, the soluble state of metabolites may be parallel to the meta-stable state of soluble proteins.

## Data accessibility

This article has no additional data.
